# Preserving Right Pre-motor and Posterior Prefrontal Cortices Contribute to Maintaining Overall Basic Emotion

**DOI:** 10.3389/fnhum.2021.612890

**Published:** 2021-02-16

**Authors:** Riho Nakajima, Masashi Kinoshita, Hirokazu Okita, Zhanwen Liu, Mitsutoshi Nakada

**Affiliations:** ^1^Department of Occupational Therapy, Faculty of Health Science, Institute of Medical, Pharmaceutical and Health Sciences, Kanazawa University, Kanazawa, Japan; ^2^Department of Neurosurgery, Faculty of Medicine, Institute of Medical, Pharmaceutical and Health Sciences, Kanazawa University, Kanazawa, Japan; ^3^Department of Physical Medicine and Rehabilitation, Kanazawa University Hospital, Kanazawa, Japan; ^4^Department of Neurosurgery, Graduate School of Medical Science, Kanazawa University, Kanazawa, Japan

**Keywords:** basic emotion, awake surgery, intraoperative monitoring, glioma, right frontal lobe

## Abstract

Basic emotions such as happiness, sadness, and anger are universal, regardless of the human species, and are governed by specific brain regions. A recent report revealed that mentalizing, which is the ability to estimate other individuals’ emotional states *via* facial expressions, can be preserved with the help of awake surgery. However, it is still questionable whether we can maintain the ability to understand others’ emotions by preserving the positive mapping sites of intraoperative assessment. Here, we demonstrated the cortical regions related to basic emotions *via* awake surgery for patients with frontal glioma and investigated the usefulness of functional mapping in preserving basic emotion. Of the 56 consecutive patients with right cerebral hemispheric glioma who underwent awake surgery at our hospital, intraoperative assessment of basic emotion could be successfully performed in 22 patients with frontal glioma and were included in our study. During surgery, positive responses were found in 18 points in 12 patients (54.5%). Of these, 15 points from 11 patients were found at the cortical level, mainly the premotor and posterior part of the prefrontal cortices. Then, we focused on cortical 15 positive mappings with 40 stimulations and investigated the types of emotions that showed errors by every stimulation. There was no specific rule for the region-emotional type, which was beyond our expectations. In the postoperative acute phase, the test score of basic emotion declined in nine patients, and of these, it decreased under the cut-off value (*Z*-score ≤ −1.65) in three patients. Although the total score declined significantly just after surgery (*p* = 0.022), it recovered within 3 months postoperatively. Our study revealed that through direct electrical stimulation (DES), the premotor and posterior parts of the prefrontal cortices are related to various kinds of basic emotion, but not a single one. When the region with a positive mapping site is preserved during operation, basic emotion function might be maintained although it declines transiently after surgery.

## Introduction

Expression of emotion and meanings of facial expressions differ according to country or area. Basic emotion (e.g., anger, disgust, fear, happiness, sadness, and surprise) is a culturally universal expression and is governed by specific brain regions (Tracy and Randles, 2011). Happiness relates to the ventral prefrontal cortex, cingulate cortex, and ventral striatum, and sadness is governed by the medial prefrontal cortex, including the anterior cingulate cortex and middle frontal gyrus (MFG), while fear mainly involves the amygdala and insula (Vytal and Hamann, 2010; Celeghin et al., 2017; Gu et al., 2019). To support this knowledge, specific emotions are damaged by focal brain damage. For instance, it has been proven in humans and animals that the sense of fear is impaired by damage to the amygdala (Adolphs et al., 1994; Calder et al., 2001; Celeghin et al., 2017), and disgust might be affected by damage to the insula and basal ganglia (Calder et al., 2000, 2001; Suzuki et al., 2006). In contrast, it has also been argued that basic emotion is not necessarily governed by a specific brain region. That is, certain brain lesions relate not only to specific emotional states but also to several kinds of neurological/neuropsychological functions (Saarimäki et al., 2016; Celeghin et al., 2017). Specifically, the amygdala and medial-prefrontal cortex play a role in various emotions and are considered to be the key brain regions for basic emotion (Phan et al., 2002; Saarimäki et al., 2016; Huang et al., 2020). As for the laterality of basic emotion in healthy people, the bilateral cerebral hemispheres are involved (Vytal and Hamann, 2010; Saarimäki et al., 2016; Gu et al., 2019). However, it is considered that the laterality of emotional function is in the right cerebral hemisphere since emotional perception is damaged more severely in right lesions than in left lesions (Kucharska-Pietura et al., 2003; Yuvaraj et al., 2013; Suslow et al., 2016; Gainotti, 2019).

Previously, attempts to monitor emotional function during awake surgery for right cerebral hemispheric gliomas have been reported. Among them, Giussani et al. (2010) first reported intraoperative monitoring of emotional perception. They assessed six types of basic emotions, namely anger, happiness, fear, surprise, disgust, and sadness, in 18 patients with right cerebral hemispheric glioma. Positive mapping sites were found at five points in five cases, and they were superior to the middle temporal gyrus to the supramarginal gyrus. Later, Herbet et al. (2015) reported intraoperative monitoring of face-based mentalizing using the Reading the mind in the eyes (RME) test (Baron-Cohen et al., 2001). The research group demonstrated that the responsible region was found in the inferior frontal gyrus (IFG). Next, to verify previous data, the same research group investigated 27 patients with low-grade glioma and found positive mapping sites in the posterior IFG, dorsolateral prefrontal cortex, and posterior superior temporal gyrus at the cortical level (Yordanova et al., 2017). Their findings corresponded to those of previous neuroimaging studies (Adams et al., 2010; Celeghin et al., 2017).

Considering the above-mentioned background, the following two hypotheses arise: (1) Does direct electrical stimulation (DES) identify the region responsible for a single basic emotion? (2) Can we preserve the ability to estimate another individual’s emotional state by preserving positive mapping sites with focal DES? Here, we demonstrated the cortical regions related to basic emotions *via* awake surgery for patients with frontal glioma and investigated the usefulness of functional mapping for preserving basic emotion. Consequently, the premotor and posterior parts of the prefrontal cortices relate to various kinds of basic emotion, but not a single emotion. When the region is preserved with the help of awake brain mapping, it might recover within 3 months postoperatively, although it declines immediately after surgery. To the best of our knowledge, our study using DES is the first to demonstrate that the right premotor and posterior prefrontal cortices are related to various types of basic emotion.

## Materials and Methods

### Participants

**Figure 1:**
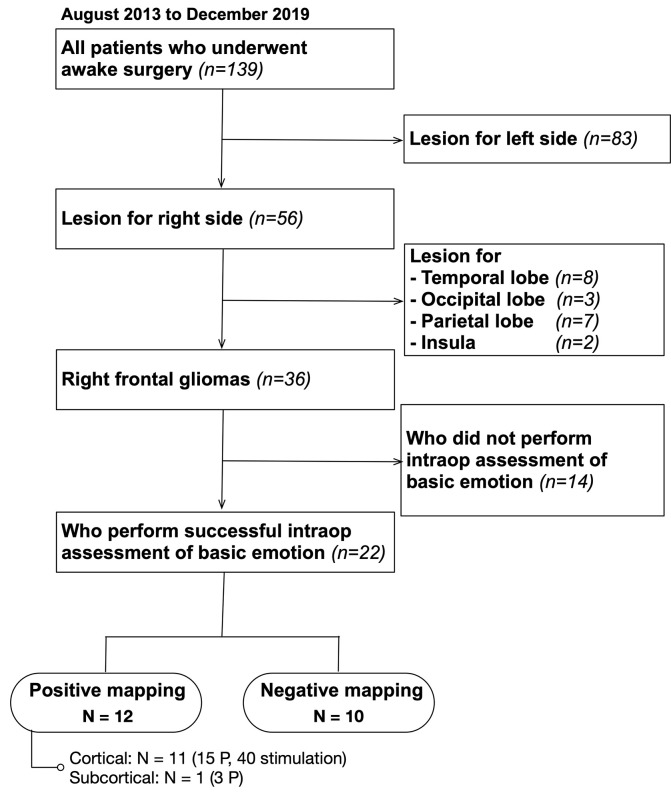
Inclusion criteria. A total of 139 patients who underwent awake surgery in our institution were included; 36 of them matched our inclusion criteria. Of these, 22 patients underwent intraoperative monitoring of basic emotion successfully and were studied. P, point.

Of the 139 patients who underwent awake surgery at our hospital between August 2013 and December 2019, 56 patients had lesions were localized on the right side. Patients in whom preoperative magnetic resonance imaging (MRI) demonstrated that lesions were found in the temporal, occipital, and parietal lobes were excluded. Finally, 36 patients with right frontal glioma were matched to our inclusion criteria (Figure 1). We decided to perform awake surgery for all patients, considering age, infiltration region, and preoperative functional condition. Of these, 22 patients underwent intraoperative assessment of basic emotion successfully (mean age: 42.8 years). Patient details are shown in Table 1. Written informed consent was obtained from all patients. This study was performed according to the guidelines of the Internal Review Board of Kanazawa University and was approved by the Medical Ethics Committee of Kanazawa University (approval numbers 1797 and 2593).

**Table 1:** Demographic and clinical characteristic of participants.

Factors	Value
Age	42.8 ± 15.6 (14–72)
Sex; male/female	10/12
Handedness; Right/left	20/2
WHO grade	I 2, II 7, III 9, IV 4
Histology	
Dysembryoplastic neuroepithelial tumor	2
Diffuse astrocytoma	2
Oligodendroglioma	4
Ependymoma	1
Anaplastic astrocytoma	2
Anaplastic oligodendroglioma	7
Glioblastoma	4
IDH-1; mutant/wild type/ND	18/3/1
1p19q; codeletion/intact/ND	10/9/1
Preop MMSE score	28.3 ± 2.0

*MMSE, mini-mental state examination; ND, not detected*.

### Awake Surgery

Awake surgery was performed using the “asleep-awake-asleep” technique, where the cortical and subcortical brain mapping was performed using DES (Duffau et al., 2002). A bipolar electrode with 5-mm-spaced tips delivering a biphasic current (pulse frequency of 60 Hz, pulse duration of 0.2 ms, amplitude between 1.5 and 6 mA, Nihon Kohden Neuromaster) was used in all cases. Patients were selected for functional monitoring during awake surgery, including visuospatial cognition, working memory, social cognition, basic emotion, and so on. In our patient group, 22 patients underwent monitoring for basic emotion. Although some reports have revealed the usefulness of functional mapping for emotional function (Herbet et al., 2015; Yordanova et al., 2017), intraoperative assessment for the neuropsychological function is still a new challenge in glioma surgery. Importantly, to achieve the best onco-functional balance for all glioma patients, we considered resection of the central part of the tumor, i.e., the enhanced lesion, and then performed intraoperative assessments on the extended resection.

To assess basic emotion intraoperatively, we used photographs of modified Japanese facial expressions of basic emotion series provided by Matsumoto and Ekman (1988). In the basic emotional test, a face of the eye region with two possible choices was presented on a computer monitor (Supplementary Figure 1). Patients were asked to select the most suitable emotional state for expressing the eyes from two choices within 2 s. Incorrect or delayed responses (≥3 s) were determined as positive responses. When positive responses were elicited in at least two out of three stimuli, the region was determined as a “positive response” and was preserved. Then, we overlaid each positive mapping site on each standardized MR image and studied the characteristics of incorrect responses during stimulation to positive mapping regions.

### Functional Assessment

All patients underwent assessment of basic emotion over time until 3 months postoperatively. As for the assessment of basic emotion, we used the expression recognition test for adults, which is most widely used in Japan (Komatsu et al., 2012). It consists of 32 photographs of basic emotions, including happiness, sadness, anger, and surprise. For each of them, a photograph with five possible choices is presented, and patients are asked to select the most reasonable mental state from the choices. Of note, since there are racial differences, assessment mainly prepared for Europeans and North Americans, such as the RME test, is not suitable for Asians (Baron-Cohen et al., 2001).

### Magnetic Resonance Imaging and Lesion Mapping

Structural MRI was performed during a 3-month postoperative period as the standard care. The images were acquired using a conventional high-resolution three-dimensional (3D) T1-weighted 3.0 T MRI scanner (Signa^™^ Excite HDx 3.0T; GE Healthcare, Little Chalfont, UK). Each MR image was reconstructed on the Montreal Neurological Institute (MNI) template using SPM12 software^1^, which runs in MATLAB^2^. We composed the resection cavities manually using the MRIcron software^3^. Each step was performed initially by RN and then systematically checked by a neurosurgeon (MK).

### Spatial Topography of Stimulations

The spatial topography of the stimulation points for emotion recognition was plotted in the following steps. First, the positive mapping sites were plotted on the corresponding original 3DT1-images for each patient using iPlan Stereotaxy 3.0 software (BrainLab), according to operative reports and intraoperative video records. The exact locations of the DES points were determined by considering their spatial relationships to various anatomical landmarks (the gyri, sulci, vessel, and midline), as previously described (Tate et al., 2014; Nakajima et al., 2020). Next, each positive mapping site of emotion recognition on the original MR images was transferred to normalized T1-images. Then, the spatial location of the positive mappings was overlaid on the 3D MNI template using the MRIcroGL software^4^. Finally, the anatomical location of positive mapping sites on the 3D MNI template was checked again in comparison to the original operative records. Each step was first undertaken by the RN and then systematically checked by a neurosurgeon (MK). For all positive mapping sites, we counted the times of stimulation for the same point (maximum, three) *via* intraoperative video recording. We then examined the types of emotions that were errors for each DES point. Besides, stimulated sites with negative responses were also determined using intraoperative multi-screen video record as the same procedure of positive mapping sites above mentioned. They were all overlapped on the 3D MNI template.

### Statistical Analysis

First, all row scores of the expression recognition test and other neuropsychological assessments were converted to *Z*-scores using the mean and standard deviations of the age-matched controls. To compare the accuracy of basic emotion recognition over time, the Steel–Dwass analysis was used. The non-parametric test was used in the current study because our patients’ data did not follow a normal distribution. All statistical analyses were performed using statistical analysis software (JMP, version 14.3.0; SAS Institute, Incorporation).

## Results

### Anatomical Data

**Figure 2:**
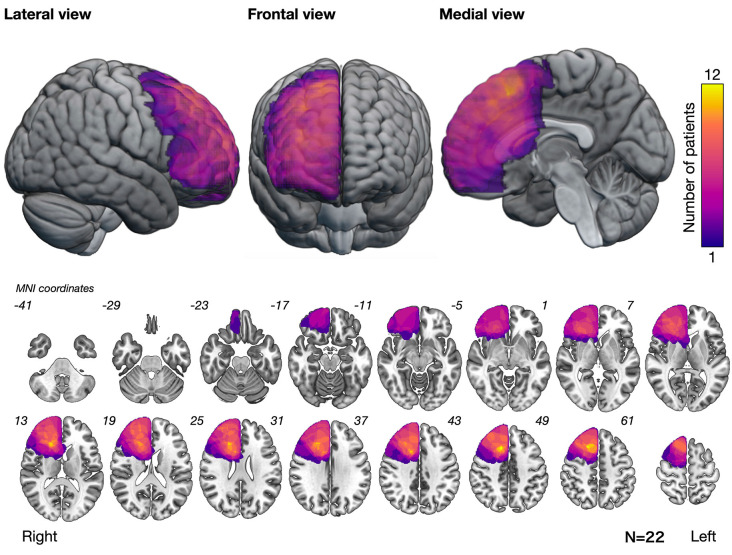
Overlap map of resection cavities showing that the posteromedial prefrontal cortex and its deep part had the greatest overlap. Numbers at the upper left of the slice images indicate the coordinates of the Montreal Neurological Institute (MNI) template.

The overlap map of resection cavities that perform an intraoperative assessment of basic emotion (*n* = 22) demonstrated that the greatest overlap was in the posterior deep white matter of the medial frontal lobe (Figure 2).

### Intraoperative Findings

**Figure 3:**
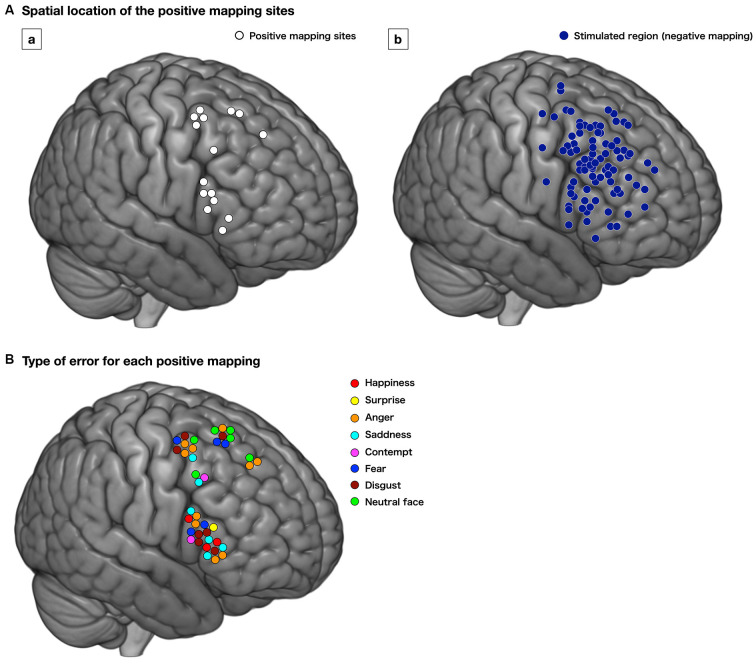
Distribution of the positive mapping sites **(Aa**, white circle) and regions with negative responses **(Ab**, dark blue circle) at the cortical level. Each positive mapping site is stimulated two or three times, and when the error or delayed responses are observed at least twice, the region is considered as “positive.” The lower image **(B)** shows the type of errors for each positive mapping. The type of emotion for which the incorrect responses were elicited is shown. Red, happiness; yellow, surprise; orange, anger; cyan, sadness; pink, contempt; blue, fear; brown, disgust; light green, neutral face.

Positive responses were found in 18 points in 12 cases (54.5%). Of these, 15 positive mappings from 11 patients were found at the cortical level, mainly the premotor and posterior part of the prefrontal cortices; with 3, 5, and 7 points for the superior frontal gyrus (SFG), MFG, and IFG, respectively (Figure 3Aa). Stimulated regions with negative responses of these patients are shown in Figure 3Ab. Then, we focused on patients whose positive mappings for basic emotion were identified during surgery in the following analysis. To note that three positive mapping sites from one patient of subcortical level, which were in the deep part of the MFG and IFG, were not investigated further in this study.

**Figure 4:**
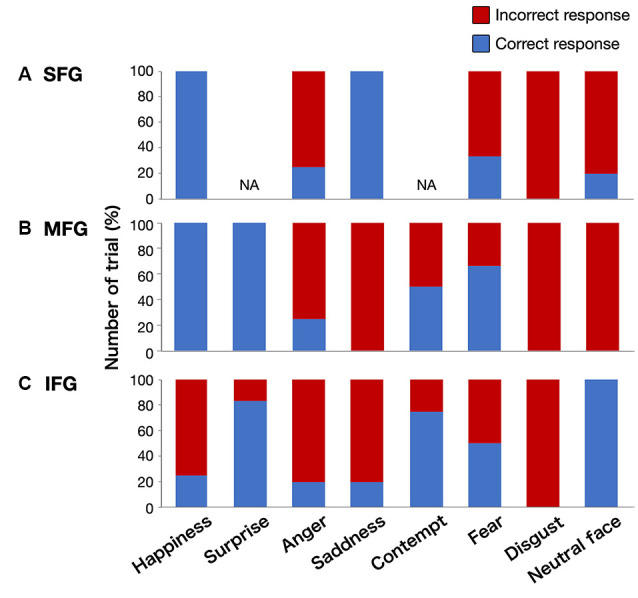
Graphs showing the percentage of correct/incorrect responses for all stimulations by emotional type. Upper column **(A)**, superior frontal gyrus (SFG); middle column **(B)**, middle frontal gyrus (MFG); lower column **(C)**, inferior frontal gyrus (IFG); NA, not applicable.

We found 56 total stimulations of positive mappings at the cortical level. Incorrect responses per total number of stimulations were 10 out of 14, 11 out of 15, and 19 out of 27 times, in the SFG, MFG, and IFG, respectively (Figure 3B). Details of patients’ incorrect responses were summarized in Supplementary Table 1. We investigated the relationship between the spatial location of incorrect responses and the emotional type and found that incorrect responses for happiness (red circle) were found only in the IFG, and errors for neutral faces (green circles) were found only in the SFG and MFG, but not in the IFG (Figures 3B, 4). In contrast, errors for sadness were found in the MFG and IFG, but not in the SFG. Also, incorrect responses were found in almost all kinds of emotions in each gyrus, and it had no other specific rule for region-emotional type.

**Figure 5:**
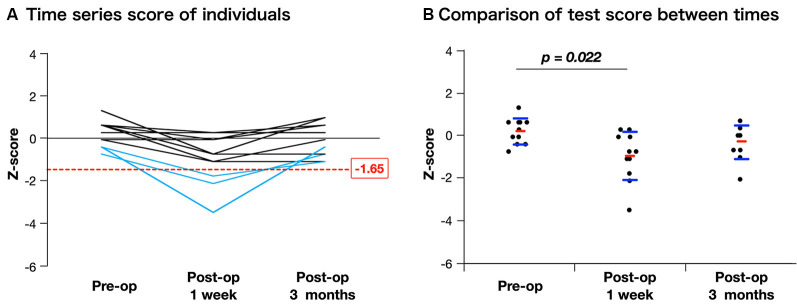
Time course of assessment for basic emotion in patients whose positive mappings were identified during awake surgery. The figure of time series of individuals **(A)** indicates that three patients declined under cut-off value (*Z* ≤ −1.65) just after surgery, but they recovered completely. When scores are compared among time series using Steel-Dwass analysis **(B)**, though the mean value declined significantly just after surgery, there is no decline at 3 months postoperatively compared with the preoperative score.

After surgery, the basic emotion test score declined in nine patients compared with the preoperative score, and of these, it declined under the cut-off value (*Z* ≤ −1.65) in three patients (Figure 5A, details are shown in Supplementary Table 2). Although the total score declined significantly just after surgery (preoperative, 0.21 ± 0.61; postoperative 1 week, −0.98 ± 1.15; *p* = 0.022), it recovered to the same preoperative level within 3 months postoperatively (−0.28 ± 0.78; Figure 5B). In our patient group, there was no postoperative significant decline of neuropsychological function such as attention or executive function which may influence the accuracy of basic emotion (Supplementary Figure 2).

### Illustrative Cases

**Figure 6:**
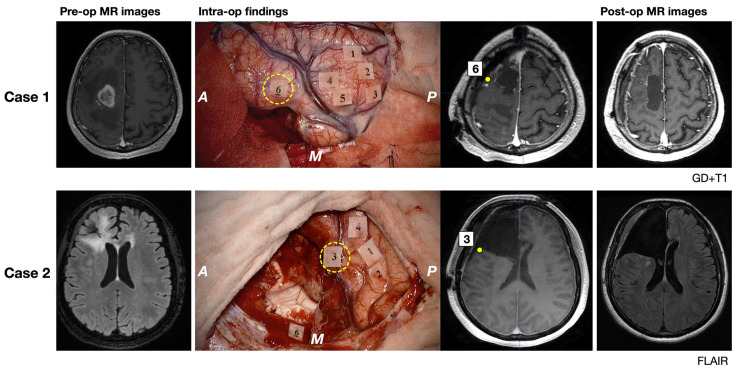
Pre- and postoperative magnetic resonance (MR) images (gadolinium-enhanced T1 image for Case 1 and FLAIR image for Case 2) and intraoperative findings of illustrative two cases are shown. MR images and number tags with the yellow circle of the middle column show the exact location of positive mappings of basic emotion identified using iPlan Stereotaxy 3.0 software (BrainLab). A: anterior; P: posterior; M: medial side; yellow broken circle, tags of incorrect responses for basic emotion. Tags and symptoms elicited by electrical stimulation are shown. Case 1: Tag 1–5, involuntary movement; Tag 6, emotion recognition; Case 2: Tag 1 and 2, dysarthria; Tag 3, emotion recognition; Tag 4, negative motor response; Tag 6, Stroop test.

Two illustrative cases are shown in Figure 6. The patient in case 1 was a 62-year-old man, whose tumor (glioblastoma, IDH-mutant) extended in the posterior pre-motor to the prefrontal area. Preoperatively, the results of neuropsychological examination revealed that attention and executive functions declined, while basic emotion was normal (*Z* = −0.41). During surgery, incorrect responses for disgust, fear, anger, and sadness were found at the posterior part of the MFG. Because incorrect responses were reproducible, the point was determined as positive mapping and it was considered to be the limit of resection. Postoperative examinations revealed that the extent of resection was 100%, and his test score for basic emotion declined under the cut-off value (*Z* = −1.79) but recovered within 3 months postoperatively. The patient in case 2 was a 39-year-old woman, whose tumor (anaplastic astrocytoma, IDH-wild type) infiltrated in the pre-motor to the prefrontal areas, and decided to undergo a second surgery. Preoperatively, a neuropsychological examination was performed, and her brain function was almost intact, except for a slight decline in working memory. She underwent awake surgery with the monitoring of basic emotion. Incorrect responses for fear, contempt, and disgust induced by DES were found at the posterior part of the IFG. Since the point was considered to be positive mapping, it was determined as the limit of resection. Postoperative MR images revealed that extended resection was performed. Postoperatively, her emotional function did not decline.

## Discussion

The primary aim of the current study was to reveal the cortical brain region related to basic emotion, and the secondary aim was to investigate the usefulness of functional mapping during awake surgery for preserving basic emotion. Consequently, the premotor and posterior parts of the prefrontal cortices are related to various kinds of basic emotion, but no frontal region is related to a single emotion. When monitoring of basic emotion is conducted during surgery and the identified localization is preserved successfully, even though the accuracy of basic emotion might be decreased temporally, it might recover. Our study is the first to demonstrate that the right premotor and posterior prefrontal cortices are related to all kinds of basic emotion, rather than a single type.

### Brain Region Relating to Basic Emotion

Through DES during awake surgery, we found that the premotor and posterior prefrontal cortices are related to various kinds of basic emotion, but we did not detect differences depending on emotional types. Previously, several studies have revealed the involvement of the premotor and prefrontal cortices in basic emotion. Indeed, the prefrontal cortex is known to be related to several kinds of emotions, such as happiness, sadness, anger, disgust, and fear (Vytal and Hamann, 2010; Dal Monte et al., 2013; Celeghin et al., 2017; Zhao et al., 2017; Gu et al., 2019). Specifically, an fMRI study demonstrated that the MFG was the common region for various kinds of facial recognition of basic emotion (Diano et al., 2017). Also, another study found that the gray matter volume of the IFG was positively related to the score of facial expression recognition for overall basic emotion (Uono et al., 2017). The mirror neuron system is also known to relate to face-based emotion recognition (Herbet et al., 2014; Perry et al., 2017). A mirror neuron is a neuron that acts selectively when observing another’s action, and it senses automatically another’s emotional state from facial expression or bodily action (Moor et al., 2012). Mirror neuron exists mainly in the premotor cortex, inferior parietal lobe, posterior superior temporal sulcus, anterior cingulate cortex, amygdala, and anterior insula (Rizzolatti et al., 1996; Lacoboni and Dapretto, 2006; Cattaneo and Rizzolatti, 2009). Among them, the premotor cortex, as well as insula considered as a core neural system for emotional processing (Jeon and Lee, 2018). Collectively, the frontal lobe is considered a key region for basic emotion (Saarimäki et al., 2016). That is, these results also indicated that the specific frontal region is not related to a single emotion (Kucharska-Pietura et al., 2003; Vytal and Hamann, 2010). Moreover, the prefrontal cortex, especially the ventral prefrontal cortex is structurally and functionally connected with the amygdala which is a center of various emotions (Li et al., 2013; Morawetz et al., 2017). The prefrontal-amygdala connectivity relates to emotion in healthy people (Clewett et al., 2014; Fernandes et al., 2019; Ibrahim et al., 2019). These findings suggest that premotor and prefrontal cortices play a role in basic emotion.

Looking in detail, the scores of sadness and disgust tend to be worse than other emotions. It is probably because of cultural differences between Caucasian-Americans and native Japanese for recognition of negative emotion (Harada et al., 2020). The Japanese are weak in expression recognition for negative emotion including sadness and disgust compared with Americans (Shioiri et al., 1999).

The positive mapping sites obtained in the current study mostly corresponded to previously reported results of intraoperative assessment of face-based mentalizing (Herbet et al., 2015; Yordanova et al., 2017). Interestingly, our study used basic emotion as an intraoperative task, while the study reported by Herbet et al. (2015) and Yordanova et al. (2017) used the RME-test, requiring patients to discriminate a more complex mental state of the face than a basic emotion. Several neuroimaging studies have reported the involvement of the right premotor and prefrontal cortices to the RME-test by several neuroimaging studies (Castelli et al., 2010; Herbet et al., 2014; Domínguez et al., 2019). This means that the premotor and posterior part of the prefrontal cortices relates to general face-based emotional perception, rather than a specific type of emotion. Taken together, assessment for all types of the emotional state rather than specific emotion might be appropriate for intraoperative monitoring at the right premotor and prefrontal regions. In contrast, a previous report revealed that the DES for the anterior insula induced response of a particular emotional type, such as anger and sadness (Motomura et al., 2019). We can take into consideration to use specific emotional type as an intraoperative assessment for the regions which induce unique emotional deficit by the DES.

### The Usefulness of DES for Preserving Basic Emotion

In the current study, basic emotion could be maintained by preserving regions with positive mappings during surgery, even though it declined immediately after surgery. This finding corresponds to that of previous studies that performed intraoperative awake mapping of emotional function (Giussani et al., 2010; Papagno et al., 2016). Additionally, the postoperative course is similar to that of several kinds of neuropsychological/neurological function assessed during awake surgery (De Witt Hamer et al., 2012; Nakajima et al., 2019).

Ideally, it is necessary to compare functional outcomes between the with- and without-intraoperative evaluation groups to determine the usefulness of awake mapping of basic emotion. However, this method is impossible because of the following reasons. First, setting a control group that does not undergo intraoperative mapping is difficult from an ethical point of view. Since, we know the advantages of intraoperative monitoring in awake conditions for several neuropsychological functions, they are still challenging (Giussani et al., 2010; Herbet et al., 2015; Papagno et al., 2016; Yordanova et al., 2017). Also, although there were no significant differences in the mean score of basic emotion between the evaluation group and non-evaluation group before surgery (Supplementary Table 3), the score of the latter declined slightly in 6 out of 14 patients (42.9%) preoperatively (*Z* ≤ −1.65, data not shown). Therefore, we decided not to use intraoperative assessment for some patients. Hence, we could not simply compare functional outcomes between the evaluation and non-evaluation groups.

Recently, the emotional function has gradually gained more attention in brain tumor surgery (Pertz et al., 2020). For instance, a report suggested that there is a high possibility of social cognition including the emotional function of patients with brain tumors being damaged preoperatively (Goebel et al., 2018). However, even now, the postoperative decline in emotional function in the right cerebral hemispheric glioma tends to be considered to recover completely in a relatively short period following surgery (Campanella et al., 2015; Mattavelli et al., 2019; Pertz et al., 2020).

In contrast, there is an important study attracting clinicians’ attention, although the number of cases is insufficient (Herbet et al., 2013). The authors investigated the postoperative course of emotion recognition in 10 patients with low-grade glioma divided into two groups according to resected lesion; those with the resected right dorsomedial prefrontal cortex and those with resected right IFG. As a result, although postoperative emotion recognition deficits in the former group were temporary, those in the latter group did not recover beyond 3 months postoperatively. Another study revealed that overall emotion recognition accuracy at a chronic phase in patients with the resected prefrontal cortex, especially the orbitofrontal cortex and medial prefrontal cortex, was significantly declined in comparison to the control group (Jenkins et al., 2014). These previous findings suggest that damage to a specific region of the frontal lobe may lead to an irreversible deficit in emotion recognition. Because the basic emotion of our patient group was maintained, in patients whose localization of basic emotion could be identified during surgery, intraoperative monitoring of basic emotion should be considered in glioma surgery for the right frontal lobe.

### Limitations

There are several limitations to the current study. First, as mentioned in the preceding section, we cannot validate the usefulness of intraoperative monitoring of basic emotion compared to the control group. Although several previous reports have revealed the utility of intraoperative monitoring for right cerebral hemispheric function including emotional function, assessment of right frontal lobe function is still a challenge (Thiebaut de Schotten et al., 2005; Herbet et al., 2015, 2016; Kinoshita et al., 2016; Nakajima et al., 2018). The usefulness of intraoperative monitoring of neuropsychological function should be considered from various aspects, such as the extent of resection, progression-free survival, overall survival, functional outcome, and postoperative social lives of patients. Another limitation is the small number of cases. However, the number of cases in previous studies on intraoperative monitoring of basic emotion was also not many; there were 5 of 18 patients with right cerebral hemispheric gliomas in Giussani et al. (2010), 13 patients with left insula glioma in Papagno et al. (2016), and 11 patients with right insula glioma who complete the intraoperative task in Motomura et al. (2019). Our study investigated the usefulness of intraoperative monitoring for basic emotion with a similar or marginally higher number of patients than the previous reports, with a focus on the right frontal lobe. Our patient’s group includes both low-grade and high-grade gliomas, although they are not biologically the same. However, several recent studies that investigated functional localization *via* awake brain mapping include both tumor types and obtain meaningful results (Gras-Combe et al., 2012; Saito et al., 2016; Nakajima et al., 2018; Puglisi et al., 2018; Rolland et al., 2018; Nakada et al., 2020). Additionally, we could not study the white matter tract, since we found only a few positive mapping sites at the subcortical level in our patient group. Third, the current study is a retrospective study of intraoperative monitoring in a clinical setting. Hence, the cortical area that can be assessed is limited to the surrounding brain tumor within the operative field. Moreover, it is unclear whether the same results can be obtained in the normal brain since slow-growing gliomas might lead to reorganization with the functional shift from original localization at the cortical level (Duffau, 2014). Although cortical reorganization for language and motor function in gliomas has been reported (Saito et al., 2016; Southwell et al., 2016; Nakajima et al., 2020), brain plasticity regarding higher brain function, such as basic emotion, is poorly understood. In support of our results, some previous reports revealed that the premotor and posterior parts of the prefrontal cortices are related to basic emotion in the normal brain (Diano et al., 2017; Uono et al., 2017). These reports showed a relatively smaller area for emotion recognition than our results. Our results might show a broader region than the original functional localization, since our data may include the region with a functional shift.

Finally, to date, the concept for basic emotion itself and localization for each emotional type is a matter of debate without consensus (Eugène et al., 2003; Touroutoglou et al., 2015; Hutto et al., 2018; Keltner et al., 2019). Further study will be required with a large number of cases to reveal which emotional states should be used in certain brain regions for intraoperative mapping, and its method for suitable assessment.

## Conclusion

The premotor and posterior parts of the prefrontal cortices are related to the different types of basic emotion. When the region with a positive mapping site is preserved, it might recover within 3 months postoperatively even though it declines immediately after surgery. Our findings will be useful in the field of neuroscience as well as for clinicians who are engaging in the treatment of brain damage, such as neurosurgery/neurology and rehabilitation.

## Data Availability Statement

The original contributions presented in the study are included in the article/Supplementary Material, further inquiries can be directed to the corresponding author.

## Ethics Statement

This study was performed according to the guidelines of the Internal Review Board of Kanazawa University and was approved by the Medical Ethics Committee of Kanazawa University (approval numbers 1797 and 2593). The patients/participants provided their written informed consent to participate in this study. Written informed consent was obtained from the individual(s) for the publication of any potentially identifiable images or data included in this article.

## Author Contributions

MN and RN: conception and design, drafting article. RN, MK, and HO: acquisition of data. RN, MK, and ZL: analysis and interpretation of data. MN: study supervision. All authors contributed to the article and approved the submitted version.

## Conflict of Interest

The authors declare that the research was conducted in the absence of any commercial or financial relationships that could be construed as a potential conflict of interest.

## Footnotes

^1^http://www.fil.ion.ucl.ac.uk/spm/software/spm12/

^2^http://www.mathworks.com/products/matlab/

^3^http://www.mccauslandcenter.sc.edu/mricro/mricron/

^4^http://www.cabiatl.com/mricrogl/

## Supplementary Material

SUPPLEMENTARY FIGURE 1 Schema of intraoperative assessment for basic emotion.

SUPPLEMENTARY FIGURE 2 These images show results of neuropsychological assessments which may influence the accuracy of emotion recognition; **(A)** Mini-mental state examination for general cognitive function; **(B)** letter cancellation test (%-correct) for selective attention; **(C)** letter cancellation test (time required) for processing speed; **(D)** Stroop test for attentional control or executive function. There were no significant differences through the course using Steel-Dwass analysis.

SUPPLEMENTARY TABLE 1 Details of patients’ incorrect responses.

SUPPLEMENTARY TABLE 2 Details of time series score of individuals.

SUPPLEMENTARY TABLE 3 Demographic and clinical factor for each group.
