# Supplementary figures and images for: Preserving Right Pre-motor and Posterior Prefrontal Cortices Contribute to Maintaining Overall Basic Emotion
Source: Front Hum Neurosci. 2021 Feb 16;15:612890. doi: 10.3389/fnhum.2021.612890 (PMC7920969; doi:10.3389/fnhum.2021.612890)

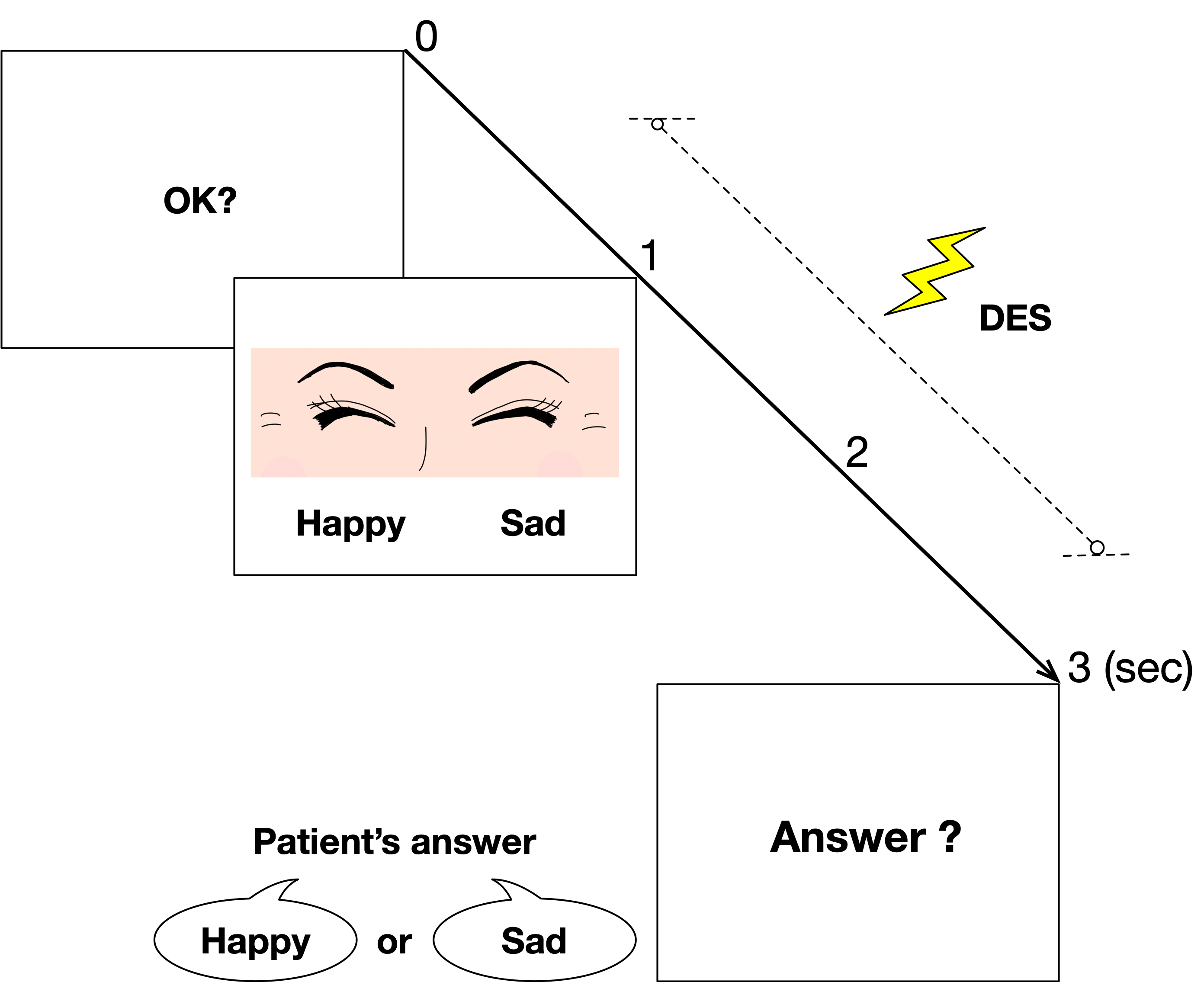

Supplement: SUPPLEMENTARY FIGURE 1 — Schema of intraoperative assessment for basic emotion. [file Image_1.TIFF]

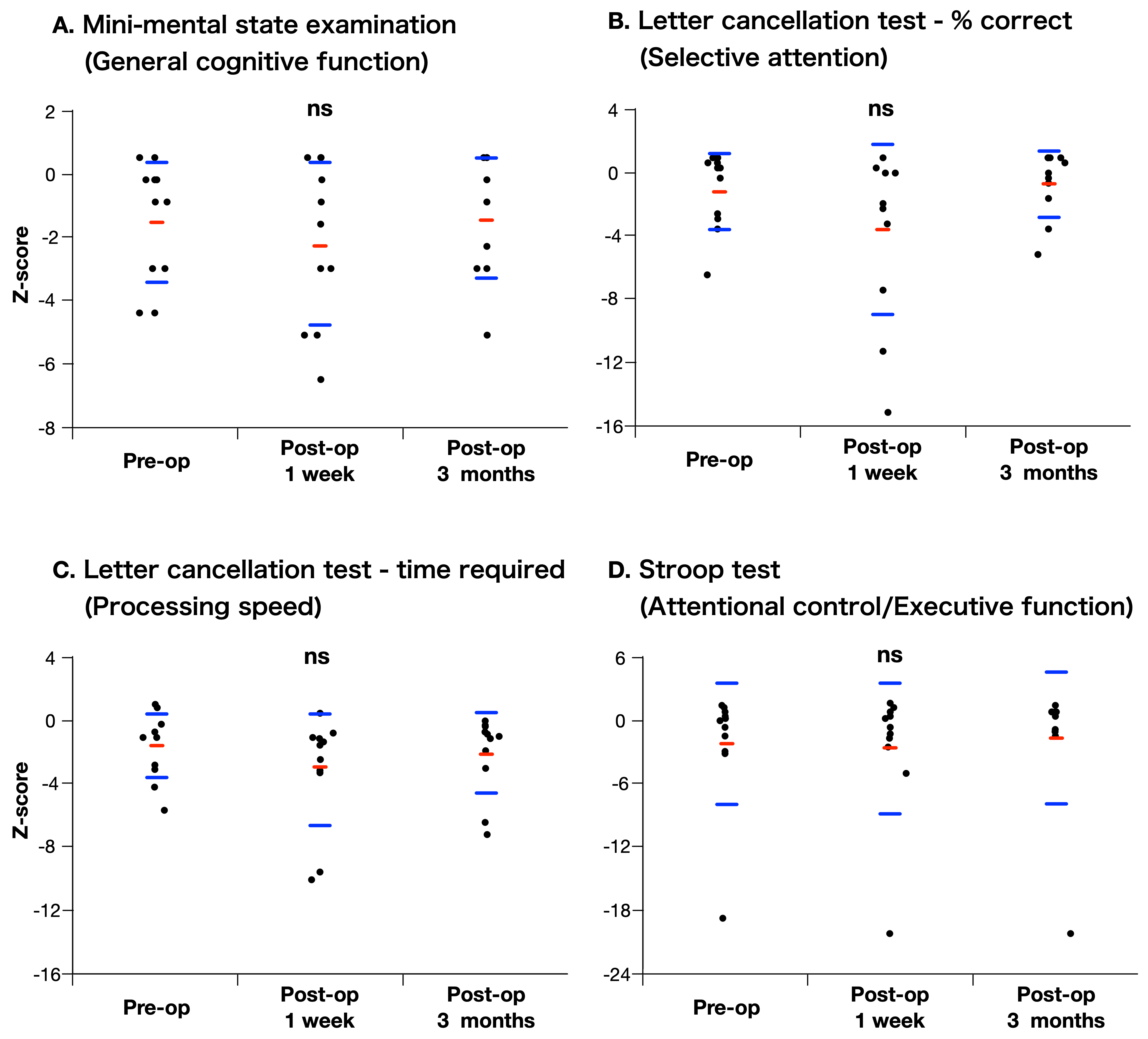

Supplement: SUPPLEMENTARY FIGURE 2 — These images show results of neuropsychological assessments which may influence the accuracy of emotion recognition; (A) Mini-mental state examination for general cognitive function; (B) letter cancellation test (%-correct) for selective attention; (C) letter cancellation test (time required) for processing speed; (D) Stroop test for attentional control or executive function. There were no significant differences through the course using Steel-Dwass analysis. [file Image_2.TIFF]
