# Supplementary material for: Preserving Right Pre-motor and Posterior Prefrontal Cortices Contribute to Maintaining Overall Basic Emotion
Source: Front Hum Neurosci. 2021 Feb 16;15:612890. doi: 10.3389/fnhum.2021.612890 (PMC7920969; doi:10.3389/fnhum.2021.612890)
Supplement: SUPPLEMENTARY TABLE 1 — Details of patients’ incorrect responses. [file Table_1.docx]

**Supplementary Table 1.** Details of patients’ incorrect responses

| **Patient** | **Stimulated region** | **Correct answer** | **Patients' response** | **Patients' incorrect response** |
| --- | --- | --- | --- | --- |
| 1 | MFG | Anger | Error | Happiness |
|  | MFG | Anger | Delay |  |
| 2 | IFG | Sadness | Delay |  |
|  | IFG | Anger | Delay |  |
|  | IFG | Happiness | Delay |  |
|  | IFG | Anger | Delay |  |
| 3 | IFG | Sadness | Error | Neutral |
|  | IFG | Anger | Delay |  |
|  | IFG | Anger | Error | Neutral |
|  | IFG | Sadness | Error | Neutral |
|  | SFG | Neutral | Delay |  |
|  | SFG | Anger | Delay |  |
|  | SFG | Anger | Delay |  |
| 4 | SFG | Neutral | Error | Sadness |
|  | SFG | Anger | Delay |  |
|  | SFG | Neutral | Error | Sadness |
| 5 | MFG | Disgust | Delay |  |
|  | MFG | Neutral | Delay |  |
| 6 | IFG | Fear | Delay |  |
|  | IFG | Contempt | Delay |  |
|  | IFG | Disgust | Delay |  |
|  | IFG | Disgust | Delay |  |
| 7 | MFG | Disgust | Error | Surprise |
|  | MFG | Fear | Delay |  |
|  | MFG | Anger | Error | Fear |
|  | MFG | Sadness | Error | Anger |
| 8 | SFG | Disgust | Delay |  |
|  | SFG | Fear | Error | Neutral |
|  | SFG | Neutral | Error | Sadness |
|  | SFG | Fear | Error | Happiness |
| 9 | IFG | Fear | Error | Neutral |
|  | IFG | Disgust | Delay |  |
|  | IFG | Surprise | Error | Contempt |
| 10 | MFG | Neutral | Delay |  |
|  | MFG | Contempt | Delay |  |
|  | MFG | Sadness | Error | Contempt |
| 11 | IFG | Sadness | Error | Anger |
|  | IFG | Happiness | Error | Contempt |
|  | IFG | Disgust | Error | Surprise |
|  | IFG | Happiness | Error | Contempt |

SFG, superior frontal gyrus; MFG, middle frontal gyrus; IFG, inferior frontal gyrus

**Supplementary Table 2**. Details of time series score of individuals

| **Age** | **Sex** | **Diagnosis** | **Z-score** | | |
| --- | --- | --- | --- | --- | --- |
|  |  |  | **Pre-op** | **Post-op**  **1 week** | **Post-op**  **3 months** |
| 23 | M | ependymoma | 0.62 | -0.07 | 0.96 |
| 63 | F | oligodendroglioma | -0.41 | -3.51 | -0.41 |
| 72 | M | oligodendroglioma | 0.62 | -1.10 | -0.07 |
| 37 | F | anaplastic astrocytoma | 0.62 | -0.76 | 0.96 |
| 47 | F | anaplastic oligodendroglioma | 0.62 | 0.28 | 0.62 |
| 43 | F | anaplastic oligodendroglioma | -0.76 | -2.13 | -0.76 |
| 28 | M | anaplastic oligodendroglioma | -0.07 | -1.10 | -1.10 |
| 59 | M | anaplastic oligodendroglioma | 1.31 | -0.76 | -0.76 |
| 35 | F | Glioblastoma | 0.28 | 0.28 | 0.28 |
| 48 | F | Glioblastoma | -0.07 | -0.07 | 0.62 |
| 62 | M | Glioblastoma | -0.41 | -1.79 | -1.10 |

M, male; F, female

**Supplementary table 3**. Demographic and clinical factor for each group

| **Factor/group** | **Non-evaluation group** | **Evaluation group** | | **P-value** |
| --- | --- | --- | --- | --- |
|  |  | **Could identify positive mapping sites** | **Could not identify positive mapping sites** |  |
| Age | 51.2 ± 12.9 | 46.5 ± 15.0 | 38.4 ± 15.9 | NS |
| WHO grade; 1/2/3/4 | 0/8/4/2 | 0/4/5/3 | 2/3/4/1 | NS |
| IDH-1 mutation; mutant/wild type/ND | 11/2/1 | 11/1/0 | 7/2/1 | NS |
| 1p19q codeletion; (-)/(+)/ND | 5/7/2 | 5/7/0 | 6/3/1 | NS |
| Pre-op tumor volume | 36.9 ± 38.1 | 56.7± 45.3 | 27.8 ± 42.6 | NS |
| Pre-op MMSE | 27.9 ± 2.3 | 27.8 ± 2.2 | 29.0 ± 1.6 | NS |
| Pre-op Emotion score (Z-score) | -0.98 ± 1.6 | 0.25 ± 0.60 | 0.55 ± 1.3 | NS |

Statistical analyses were performed to compare each factor among three groups using Steel-Dwass test. NS, not significance; MMSE, Mini-mental state examination.
